# Boosting Ru atomic efficiency of LaFe_0.97_Ru_0.03_O_3_*via* knowledge-driven synthesis design†

**DOI:** 10.1039/d5sc00778j

**Published:** 2025-03-28

**Authors:** Yu Wang, Paul Paciok, Lukas Pielsticker, Alexander Spriewald Luciano, Lorena Glatthaar, Aijie Xu, Zimo He, Min Ding, Walid Hetaba, Jaime Gallego, Yanglong Guo, Bernd M. Smarsly, Herbert Over

**Affiliations:** a State Key Laboratory of Green Chemical Engineering and Industrial Catalysis, Research Institute of Industrial Catalysis, School of Chemistry and Molecular Engineering, East China University of Science and Technology Shanghai 200237 PR China ylguo@ecust.edu.cn; b Institute of Physical Chemistry, Justus Liebig University Heinrich-Buff-Ring 17 D-35392 Giessen Germany Herbert.Over@phys.Chemie.uni-giessen.de Bernd.Smarsly@phys.Chemie.uni-giessen.de; c Ernst Ruska-Centre for Microscopy and Spectroscopy with Electrons and Peter Grünberg Institute, Forschungszentrum Jülich GmbH Jülich 52425 Germany; d Department of Heterogeneous Reactions, Max Planck Institute for Chemical Energy Conversion Stiftstr. 34-36 45470 Mülheim an der Ruhr Germany; e Center for Materials Research, Justus Liebig University Heinrich-Buff-Ring 17 D-35392 Giessen Germany

## Abstract

The exsolution of ruthenium from a 3 at% ruthenium-substituted LaFeO_3_ (LFR3) perovskite oxide is meticulously designed to produce a high-performance ruthenium-supported catalyst with high atomic efficiency. A high-temperature redox pretreatment at 800 °C enriches the Ru concentration in the near-surface region of LFR3, while a subsequent mild reduction step with H_2_ at 500 °C leads to the Ru exsolution from the Ru-enriched near-surface region (LFR3_Redox_500R), resulting in a high density of small particles that are not passivated by LaO_*x*_. The performance of this catalyst is evaluated through its application in two prototypical catalytic reactions: the combustion of propane (oxidation reaction) and the reduction of CO_2_ by hydrogenation (reduction reaction). For both reactions, the activity of the redox-pretreated sample LFR3_Redox_500R exhibits a significant increase compared to the activity of the untreated sample (LFR3_500R). In the catalytic hydrogenation of CO_2_, the high selectivity profile undergoes a transition from CO for LFR3_500R to methane for LFR3_Redox_500R.

## Abstract 2

The exsolution of ruthenium from a 3 at% ruthenium-substituted LaFeO_3_ (LFR3) perovskite oxide is meticulously designed to produce a high-performance ruthenium-supported catalyst with high atomic efficiency.

## Graphical abstract

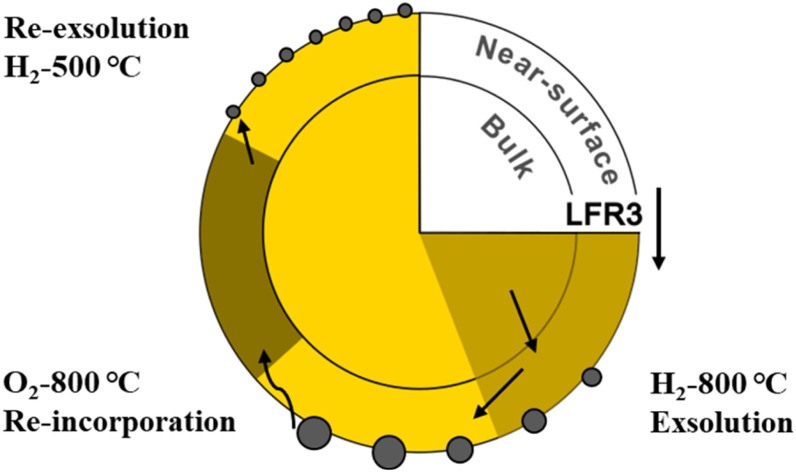

## 1 Introduction

Instead of depositing catalytically active nanoparticles on a support material, as in the conventional “top-down” process, the “bottom-up” approach, termed exsolution, has become a powerful platform for the design of advanced materials in catalysis by a controlled phase separation of a homogeneous solid oxide solution.^1–6^ The active component is first doped into the host oxide lattice during preparation and then exsolved as nanoparticles on the surface by high-temperature reduction, resulting in a narrow size distribution of anchored (socketed) small particles.^2,7–9^ Therefore, exsolved socketed nanoparticles show great resistance to the particle agglomeration and sintering during high-temperature reactions such as those encountered in the anode reaction of solid electrolyte fuel cells^10–14^ and ammonia synthesis, while maintaining an optimal Ru particle size of 5 nm during the reaction.^15^

A particularly useful feature of exsolved nanoparticles is their propensity to be re-dissolved into the parent support lattice by the application of high-temperature oxidation so that subsequently the active component can be re-exsolved to restore the original catalyst morphology. This phenomenon is referred to as “self-regeneration”,^16^ which may represent an “intelligent catalyst”,^17^ as it offers the unique opportunity to regenerate a catalyst *in situ* after deactivation by sintering, poisoning, coking *etc.* has occurred. However, depending on the defect chemistry of the host oxide, the regeneration process is asymmetric in that the reductive exsolution process is much faster and more efficient than the oxidative redissolution process.^18,19^ This asymmetry in redox behavior may result in partial reincorporation of the active component into the host lattice,^18,20,21^ or the exsolution/dissolution process may not be reversible at all.^22^

Perovskite oxides (ABO_3_) are often chosen as the starting material for exsolution because of their stability and structural flexibility.^23,24^ Both the A and B sites allow for the doping with a wide variety of different cations.^25^ The active component to be exsolved from ABO_3_ is typically substituted in the B sites and is easier to chemically reduce than the substituted B site element. This allows selective exsolution of the active component.^1,26^

Exsolution is a multi-step process consisting of metal ion diffusion to the surface, reduction of the metal ion to the metal, followed by nucleation and growth of the particles on the surface.^27,28^ In some cases, the exsolution of the target element is accompanied by undesired segregation of other elements from A and B sites, which affects the catalytic performance.^29,30^ The ultimate goal of the catalyst preparation is to achieve a high concentration of small and stable particles of the active component on the support surface that are active in the catalytic reaction under consideration.

However, the high calcination temperature during the preparation of perovskite oxides results in the formation of large oxide particles so that the exsolution of the active component preferentially occurs from the near-surface region of the perovskite. As a result, significant amounts of the active component remain buried in the bulk of the perovskite particle after the exsolution process and do not contribute to the catalytic activity of the material, resulting in a low atomic efficiency of the catalyst. High-temperature redox treatment has been shown to increase the near-surface concentration of the active component by diffusion from the bulk.^31^ This strategy shows promise in enhancing the atomic efficiency of the exsolution process.

Ruthenium-substituted LaFeO_3_ (LFRO, perovskite) has been reported to have an additional problem, namely the formation of a passivating LaO_*x*_ layer on the exsolved particles during high-temperature reduction at 800 °C, which reduces the catalytic activity.^32^ A recent study has shown that the exsolution of Ru begins at 450–500 °C, while the formation of the LaO_*x*_ layer commences at 600 °C.^33^

With this detailed information in mind, we devise here a strategy to obtain an improved exsolution catalyst with high atomic efficiency. We start with LFR3 in which 3 at% of the Fe position is substituted by Ru, corresponding to 1.3 wt% of ruthenium, which is a typical loading of a supported catalyst when using noble metals as the active component. To increase the mass activity of Ru in LFR3, *i.e.*, the activity per gram of Ru, the ruthenium concentration in the near surface region is first enriched by a high temperature redox step at 800 °C (LFR3_Redox). The ruthenium is then exsolved under reducing conditions at 500 °C, a temperature that is below the onset of LaO_*x*_ layer formation, forming small Ru particles with an average size of less than 2 nm, resulting in a high dispersion (LFR3_Redox_500R). In this way it is possible to produce a highly active catalyst which is not passivated by a LaO_*x*_ top layer and whose mass activity is increased by a factor of about four when compared to the exsolved LFRO_500R catalyst without prior high-temperature redox pretreatment. Catalytic tests are performed with an oxidation reaction (propane combustion) and a reduction reaction (CO_2_ reduction by H_2_). For both reactions the catalyst with redox pretreatment LFR3_Redox_500R is shown to be active and stable, with better catalytic performance than LFR3_500R.

## 2 Materials and methods

### 2.1 Samples preparation

The Ru-doped perovskite is prepared by the Pechini method using citric acid as a chelating agent.^34^ Specifically, 4 mmol of La(NO_3_)_3_·6H_2_O, 3.88 mmol of Fe(NO_3_)_3_·9H_2_O, 0.12 mmol of Ru(NO)(NO_3_)_3_, 12 mmol of citric acid and 24 mmol of ethylene glycol are dissolved in 50 ml of distilled water. A brown gel gradually forms as the water evaporates when the flask is placed in an oil bath at 80 °C. After drying overnight at 120 °C, the gel is ground and transferred to the crucible. To obtain the perovskite oxide, the sample is calcined in a muffle furnace at 300 °C for 1 h and 750 °C for 3 h at a ramp rate of 3 °C min^−1^. The resulting black powder (nominally LaFe_0.97_Ru_0.03_O_3_) is designated LFR3. The high-temperature redox treated sample LFR3_Redox is prepared by reduction of LFR3 at 800 °C for 3 hours in 4 vol% H_2_/Ar that is followed by calcination in static air at 800 °C for 3 h. Finally, the as-synthesized LFR3 and LFR3_Redox samples are reduced in a tube furnace under 4 vol% H_2_ balanced by Ar (flow rate of 100 ml min^−1^) at 500 °C for 3 h to exsolve the Ru particles. The resulting samples are referred to LFR3_500R and LFR3_Redox_500R, respectively.

### 2.2 Samples characterizations

The structural and textural characterization of the perovskite oxides is performed using a Panalytical X'Pert PRO diffractometer equipped with a Cu Kα radiation source (*λ* = 1.5418 Å) operating at 40 kV and 40 mA. The powder diffraction patterns of the perovskite oxides are scanned with a step size of 0.013° over a range of 2*θ* from 20–75° at a rate of 200 s per step. The Ru content in the Ru-doped perovskite oxides is quantified by inductively coupled plasma-atomic emission spectrometer (ICP-AES) on a PerkinElmer Optima 2100 DV spectrometer. Raman measurements are performed using a Bruker Optics Senterra spectrometer with a laser wavelength of 532.8 nm at a power of 2 mW. All samples are measured with a spectral resolution of 5 cm^−1^, 200 co-additions, and an integration time of 8 seconds. The Raman spectra are recorded in the backscattering geometry at room temperature and analyzed using the OPUS 7.5 software. The surface areas are measured on Micromeritics ASAP 2020M operating at −196 °C. Prior to the BET experiments, catalysts are degassed at 180 °C for 12 h.

The composition and chemical environment of Ru in the near surface region are analyzed by *ex situ* X-ray photoelectron spectroscopy using a PHI 5000 VersaProbe II instrument (Physical Electronics GmbH) with Al Kα radiation (1486.7 eV). For the data processing, the peak positions of the spectra are calibrated by the adventitious carbon at a binding energy of 284.8 eV. The exsolution process of Ru is also followed by *in situ* near ambient pressure X-ray photoelectron spectroscopy (NAP-XPS, Specs GmbH) equipped with a Phoibos NAP-150 hemispherical analyzer and with a monochromatic Al Kα source. The perovskite oxides are first pretreated in 0.5 mbar O_2_ at 600 °C for 30 min to remove the adventitious carbon species on the surface. H_2_ is then introduced into the sample chamber under mass flow control, maintaining the pressure at 1 mbar during the measurement. The sample is held at each temperature (300, 400, 500 °C) for 1 h, after which the XP spectra are collected. The XPS spectra are analyzed using Casa XPS version 2.3.17 software.

The morphology of the analyzed samples is visualized by scanning transmission electron microscopy (STEM, Thermo Fisher Talos F200X microscope) at an accelerating voltage of 200 kV. EDS mapping is performed using 4 in-column Super-X detectors. Scanning electron microscopy (SEM) is conducted on a MERLIN instrument (Carl Zeiss AG, Germany) to obtain the morphology of LFR3_800R. Density and size of the Ru-exsolved samples are measured in another STEM (Hitachi HF5000) coupled with secondary electron detector at 200 kV operating voltage. After suspending the perovskite powder in the ethanol, a drop is deposited onto a Cu grid with a carbon film.

Temperature programmed H_2_ reduction (H_2_-TPR) experiments are performed on a PX200 instrument coupled to a TCD detector. 50 mg of the Ru-doped perovskites are placed in a U-shaped quartz tube and prior to the H_2_-TPR experiments, the sample is pretreated in Ar (40 ml min^−1^) at 400 °C for 30 min to clean the surface. The sample is then heated from room temperature to 600 °C at a ramp rate of 10 °C min^−1^ in 10 vol% H_2_/N_2_. H_2_ uptake values for the reduction peak are calibrated using the TPR profile of CuO.

The content of exposed Ru in the Ru-exsolved samples are determined by a CO uptake experiments performed at room temperature on a Micromeritics Auto chem II 2920 chemisorption coupled to an HPR-20 QIC benchtop mass spectrometer. 50 mg of the sample is placed in a U-shaped quartz tube and pretreated with 10 vol% H_2_/Ar at 400 °C for 30 min at a flow rate of 40 sccm. After cooling the sample to room temperature, 1 vol% CO/He is injected into the reactor every two minutes until the CO signal at the outlet returns to the baseline. Assuming that the ratio of CO to Ru is one, the number of the active sites can be calculated. The dispersion is defined as the ratio of the number of exposed surface Ru sites as derived from CO uptake experiments to the total amount of Ru in the sample.^10^

*In situ* diffuse reflectance infrared Fourier transform spectroscopy (DRIFTS) of CO adsorption is performed in a Bruker Vertex 70V spectrometer coupled to an MCT detector.^35^ The powder sample is ramped to 300 °C in 4 vol% H_2_/Ar at a flow rate of 50 sccm for 1 h, then the atmosphere is changed to pure Ar while maintaining the same flow rate. After cooling the sample to room temperature, 2 vol% CO/Ar is introduced into the cell for 30 min to reach a stable conditions for DRIFT spectra collection. The intensity of the gaseous CO Ro-Vi band is used as a reference to normalize the intensity of the DRIFT spectra.

### 2.3 Catalytic activity evaluation

The catalytic performance of the investigated perovskite samples in the total oxidation of propane is carried out in a home-built fixed-bed reactor.^36^ 20 mg of the perovskite sample diluted with 40 mg of inert quartz sand is loaded into a quartz tube with an inner diameter of 6 mm. During the activity measurement, the sample is heated in the reaction mixture (1 vol% C_3_H_8_, 10 vol% O_2_ and 89 vol% N_2_) at a total flow rate of 100 sscm from room temperature to 400 °C at a ramp rate of 1 °C min^−1^, reaching a weight hourly space velocity (WHSV) of 34 500 ml g^−1^ h^−1^. The effluent gas is analyzed with a non-dispersive infrared sensor (Saxon Junkalor INFRALYT 80) to quantify the concentration of C_3_H_8_ and CO_2_ according to Beer–Lambert law since they have different characteristic absorption bands. The C_3_H_8_ conversion is calculated using eqn (1):1
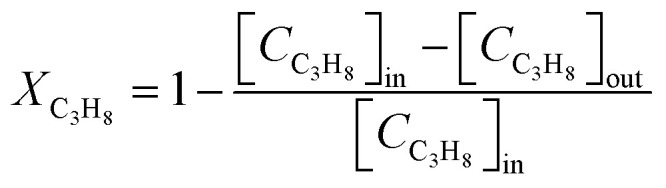
where the [*C*_C_3_H_8__]_in_ and [*C*_C_3_H_8__]_out_ stand for the C_3_H_8_ concentration in the inlet and outlet gas, respectively.

The kinetic data for the propane oxidation reaction is tested in the same reaction condition, with the propane conversion limited to 10% to avoid heat transfer limitations and to stay in the kinetic regime. The space-time yield (STY) is defined as the molar amount of product per time and per mass of catalyst, expressed as mol_CO_2__ h^−1^ kg_cat_^−1^. To determine the intrinsic activity of the samples, the STY value is normalized to the number of active sites derived from the CO uptake experiment to obtain STY_*n*_, which is given in the unit of mol_CO_2__ mol_active site_^−1^ s^−1^.

The CO_2_ hydrogenation is carried out in a fixed bed reactor at atmospheric pressure. 4 sccm of CO_2_ and 16 sccm of H_2_ are balanced with 20 sccm of N_2_ as an internal reference and fed into the reactor containing 100 mg of the catalysts. Prior to the activity test, the sample is pretreated *in situ* in 4 vol% H_2_/Ar at 500 °C for 30 min. After cooling down the sample to room temperature, the feed gas is switched to the reactant gas. The conversion curve is measured over the temperature range from 200 °C to 500 °C. To determine the conversion and selectivity, the effluent gas is analyzed on an Agilent 7890A gas chromatograph using the TCD detector coupled to a TDX-01 column to quantify the CO_2_ and CO concentrations. The FID is coupled to a HP-PLOT Q capillary column to monitor CH_4_ and other organic products. Due to the negligible amounts of other products, only CO and CH_4_ are considered in the selectivity calculation. The CO_2_ conversion and the selectivity are determined using eqn (2)–(4)2
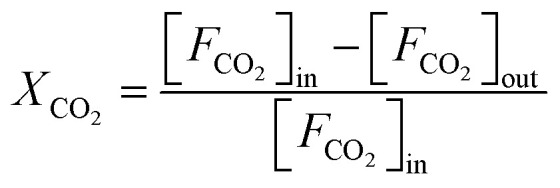
3
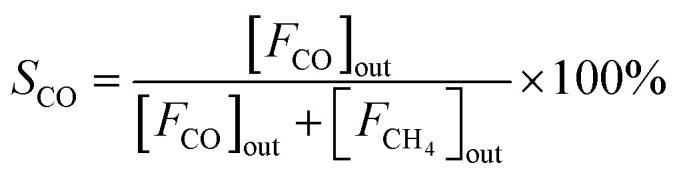
4
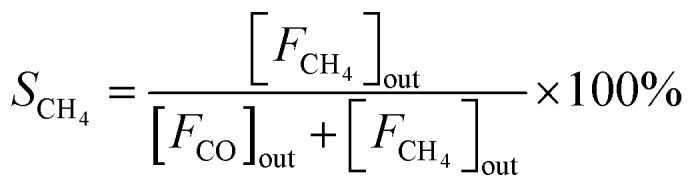
where [*F*_CO_2__]_in_ represents the flow rate of CO_2_ in the inlet gas while [*F*_CO_2__]_out_, [*F*_CO_]_out_ and [*F*_CH_4__]_out_ means the flow rate of CO_2_, CO and CH_4_ in the outlet gas, respectively.

## 3 Results and discussions

### 3.1 Experimental results

**Fig. 1:**
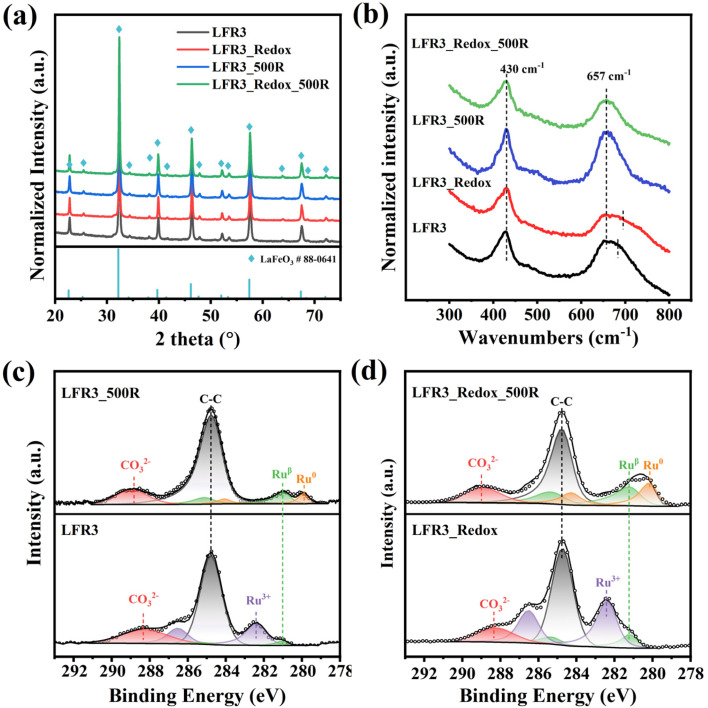
(a) XRD patterns (blue diamonds are the position of orthorhombic LaFeO_3_) and (b) Raman of the Ru-incorporated samples LFR3, LFR3_Redox and Ru-exsolved samples LFR3_500R, LFR3_Redox_500R. Fitted C 1s + Ru 3d *ex situ* XPS spectra of (c) LFR3, LFR3_500R and (d) high-temperature redox treated samples LFR3_Redox and LFR3_Redox_500R.

The X-ray diffraction (XRD) patterns for the Ru-doped and exsolved perovskite oxides are summarized in Fig. 1a. For the as-synthesized LFR3, all the diffraction peaks can be traced to the characteristic patterns of orthorhombic LaFeO_3_ (JCPDS No. 88-0641), indicating the successful preparation of the perovskite structure. For the high-temperature redox-treated sample LFR3_Redox, virtually identical XRD results indicate that the perovskite structure is preserved. Furthermore, the XRD patterns of these two samples reveal no significant changes after reductive treatment at 500 °C, probably due to the relatively small particle size of exsolved Ru, which is below the detection limit of XRD. All samples have similar specific surface areas below 10 m^2^ g^−1^ (Table 1). Combined with the XRD results, this suggests that no significant structural changes occur in these Ru-containing perovskite oxides.

**Table 1:** The physical and chemical properties of the Ru-containing perovskites LFR3 and LFR3_Redox and after mild reduction at 500 °C: LFR3_500R and LFR3_Redox_500R

Samples	*S*_BET_ a (m^2^ g^−1^)	Ru content^b^ (wt%)	Ru content^c^ (at%)	Ru size^d^ (nm)	Particle density^d^ (μm^−2^)	Dispersion^e^*D*_Ru_ (%)	H_2_ consumption^f^ (μmol g_cat_^−1^)	
							≤500 °C	>500 °C
LFR3	8	1.3	2.4	—	—	—	146	61
LFR3_500R	7	—	2.4	2.26	4350	8.2	—	—
LFR3_Redox	9	1.3	6.7	—	—	—	239	56
LFR3_Redox_500R	7	—	6.4	1.85	26 000	25.6	—	—

a Determined by BET method.

b Measured by ICP-AES.

c Derived by *ex situ* XPS.

d Assessed by SE-STEM.

e Calculated by CO uptake experiment.

f Quantified based on H_2_-TPR experiments using CuO as the reference.

Raman spectroscopy (*cf.*Fig. 1b) has been demonstrated to provide valuable insights into the exsolution process and the bulk properties of LFR3 based samples prior to and following reduction. All four samples show similar Raman features known for orthorhombic LaFeO_3_ (Fig. S1†). The substitution of 3% Fe by Ru results in the distortion of the FeO_6_ octahedron, which is associated with a broadening of the O–Fe–O bending mode at 433 cm^−1^, a slight blue shift of the peak C from 643 cm^−1^ to 657 cm^−1^, and the appearance of a shoulder around 670 cm^−1^.^37^ The shoulder broadens further after high temperature redox treatment. However, the shoulder around 670 cm^−1^ disappears in the Raman spectra of LFR3 and LFR3_Redox after reductive treatment at 500 °C, probably due to the exsolution of Ru particles.

X-ray photoelectron spectroscopy (XPS) in Fig. 1c is able to determine the chemical state and atomic ratio of Ru in the near-surface region. The spectral feature at 289 eV in the (Ru 3d + C 1s) spectra is attributed to carbonate species resulting from CO_2_ adsorption on the lanthanum oxides.^38^ The Ru 3d XP spectrum of LFR3 is predominantly characterized by a Ru^3+^ feature which is associated with the Ru on the B site of the perovskite structure, substituting for Fe^3+^. This finding indicates that the Ru is successfully doped into the perovskite oxide lattice.^32,39^ In addition, a small Ru^β^ feature emerges with a binding energy of 281.1 eV, corresponding to Ru species in a lower oxidation state than +3 that is likely coordinated to oxygen vacancies.^33^ As shown in Fig. S2,† following reductive treatment at 800 °C in 4 vol% H_2_ for 3 h, the Ru^3+^ signal completely disappears and instead Ru^0^ becomes the dominant state of ruthenium. This observation indicates that a significant portion of the Ru present in the near-surface region of LFR3 is exsolved. This hypothesis is further substantiated by the presence of some nanoparticles, as evidenced by SEM analysis (*cf.* Fig. S3a†). The missing Fe^0^ signal in Fig. S4† suggests that there is no noticeable Fe exsolution, while the hysteresis between first two reaction cycles in Fig. S5† can be attributed to the formation of inert LaO_*x*_ coating layer on LFR3_800R. The complete reincorporation of exsolved Ru upon reoxidation of LFR3_800R at 800 °C for 3 hours (observed in SEM: LFR3_Redox, Fig. S3b†) is also confirmed by the reappearance of the Ru^3+^ and the disappearance of the Ru^0^ feature in the Ru 3d spectrum (*cf.*Fig. 1d). In principle, Ru could have been oxidized to volatile RuO_3_ and RuO_4_ at temperatures higher than 600 °C.^40^ However, the total Ru content in the sample remains unchanged after the redox treatment (as determined by ICP-AES), indicating that all the ruthenium in the exsolved Ru particles dissolves back into the perovskite lattice. It is noteworthy that the intensity of the Ru 3d signal in the LFR3_Redox XP spectrum is considerably higher than that in LFR3. *Ex situ* XPS data analysis reveals that the total amount of Ru in LFR3_Redox is 6.8 at%, which is approximately three times higher than that of the as-synthesized LFR3 sample (2.4 at%). When LFR3 and LFR3_Redox are reduced at 500 °C for 3 hours in 4 vol% H_2_/Ar, the Ru^3+^ signal in the Ru 3d spectra disappears and is replaced by Ru^β^ and Ru^0^ (due to metallic ruthenium in the exsolved particle) as a result of the continuous reduction of Ru^3+^: Ru^3+^ → Ru^β^ → Ru^0^.^33^

**Fig. 2:**
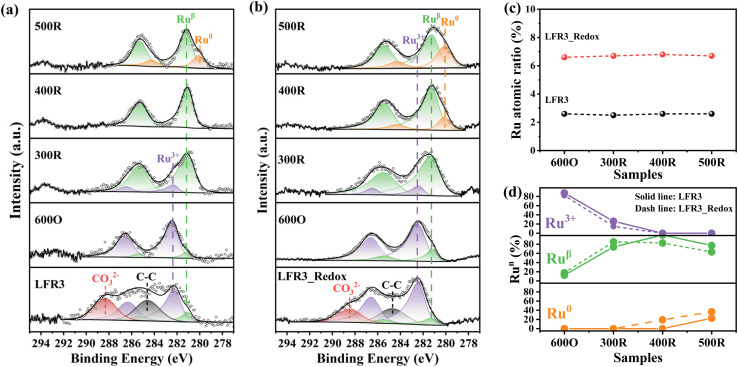
Fitted C 1s + Ru 3d NAP-XPS of (a) the as-loaded LFR3 and (b) LFR3_Redox, carbon elimination after oxygen pretreatment at 0.5 mbar O_2_ and 600 °C for 1 h (600O) and various reduction treatment at different temperatures (300 °C, 400 °C and 500 °C) under 1 mbar H_2_ for 1 h (300R, 400R, 500R). (c) The derived total Ru content and (d) the fraction of Ru in different oxidation states. The fitting parameters are provided in Tables S1 and S2.†

In order to elucidate the Ru exsolution process of LFR3 and LFR3_Redox during the reduction treatment, near ambient pressure X-ray photoelectron spectroscopy (NAP-XPS) experiments of LFR3 and LFR3_Redox are performed under 1 mbar H_2_ at different temperatures. The fitted C 1s and Ru 3d spectra are summarized in Fig. 2, while the derived compositions are compiled in Table 2. Prior to the *in situ* reduction experiment, the samples undergo a pre-treatment in 0.5 mbar O_2_ at 600 °C for 1 h to remove the majority of the C 1s related features from the Ru 3d spectra. The Ru 3d5/2 spectrum exhibits three prominent signals with binding energies of ∼282.5 eV, ∼281.2 eV, and ∼280.1 eV, which are attributed to Ru^3+^, Ru^β^, and Ru^0^, respectively. When exposed to a reductive environment at 300 °C, Ru^3+^ is partially converted to Ru^β^, with the result that Ru^β^ begins to dominate the Ru 3d spectra for both LFR3 and LFR3_Redox samples. Increasing the reduction temperature to 400 °C results in a complete conversion of Ru^3+^ to Ru^β^ in LFR3, while in LFR3_Redox Ru^3+^ converts completely to Ru^β^ and Ru^0^ with molar fractions of 85% and 15%, respectively (Fig. 2b and d).

**Table 2:** NAP-XPS-derived near-surface composition and Ru oxidation state distribution of LFR3 and LFR3_Redox after pretreatment in 0.5 mbar oxygen and different temperatures from 300 °C to 500 °C in 1 mbar H_2_ for 1 h

Samples	LFR3^a^						LFR3_Redox^a^					
	Atomic ratios^a^ (%)			Ru^*n*^ (%)			Atomic ratios^a^ (%)			Ru^*n*^ (%)		
	Ru	La	Fe	Ru^3+^	Ru^β^	Ru^0^	Ru	La	Fe	Ru^3+^	Ru^β^	Ru^0^
_600O	2.6	50.9	46.5	87.6	12.4	0	6.7	58.5	34.8	83.0	17.0	0
_300R	2.5	50.7	46.8	25.0	75.0	0	6.6	55.5	37.9	14.8	85.2	0
_400R	2.6	50.8	46.6	0	100	0	6.8	59.4	33.8	0	82.6	17.4
_500R	2.6	54.1	43.3	0	74.2	25.8	6.7	56.6	36.7	0	64.2	35.8

a Determined by *in situ* XPS.

At a reduction temperature of 500 °C, Ru^0^ species appear in the XP spectrum of LFR3 XP with 75% Ru^β^ and 25% Ru^0^. In contrast, for the LFR3_Redox sample, the intensity of the Ru^0^ signal increased substantially after reduction at 500 °C, accounting for 36% of the Ru 3d spectral area. These observations suggest that LFR3_Redox exhibits a higher propensity to form metallic Ru under less extreme reduction conditions compared to LFR3. The molar fractions of Ru^3+^, Ru^β^, and Ru^0^ during the reduction treatment of LFR3 and LFR3_Redox are outlined in Fig. 2d. The observation that the total Ru content in both LFR3 and LFR3_Redox remains constant during the reduction process up to 500 °C (*cf.*Fig. 2c) suggests that the Ru exsolution is limited to the near-surface region. As illustrated in Fig. S6,† there are no obvious changes on the binding energy of LFR3 and LFR3_Redox after reduction treatment at 500 °C, which evidences that La has not been segregated after the reduction treatment at this mild temperature.

As illustrated in Fig. S7,† high angle annular dark field (HAADF) images of the samples reveal that the surfaces of the Ru-incorporated samples LFR3 and LFR3_Redox (Fig. S7a and b†), appear to be relatively smooth. This observation suggests that Ru is incorporated into the perovskite lattice rather than segregating into separate large Ru particles. However, upon reduction of LFR3 and LFR3_Redox at 500 °C, the presence of some particles on the surface is observed (Fig. S7c and d†). The appearance of Ru^0^ in XPS (Fig. 1c, d and 2) suggests the newly formed particles are exsolved Ru. Energy dispersive X-ray spectroscopy (EDS) in the right panels of Fig. S7c and d† confirms the composition of these particles as Ru, thereby demonstrating the successful exsolution of Ru by the reductive treatment at 500 °C. However, the low contrast of the images hinders the accurate quantification of the particle density and size distribution remains a challenge.

**Fig. 3:**
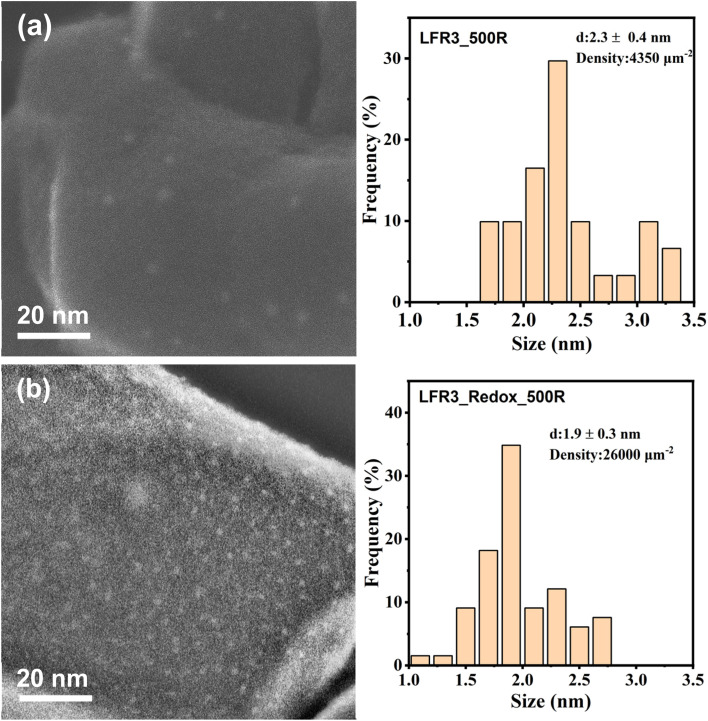
Secondary electron (SE)-STEM images of (a) LFR3_500R and (b) LFR3_Redox_500R. The corresponding size distributions are presented in the right panel. The STEM images are representative.

Secondary electron STEM imaging (*cf.*Fig. 3), which provides high surface sensitivity, highlights the surface topography and allows for the quantification of the particle size and distribution. The average size of exsolved Ru of LFR3 is found to be 2.3 nm which is slightly larger than that of LFR3_Redox_500R (1.9 nm). However, the particle density is significantly different in these samples. For LFR3_500R, the particle density is estimated to be approximately 4350 μm^−2^, which is considerably lower than that of the high-temperature redox pretreated sample LFR3_Redox_500R with a particle density of approximately 26 000 μm^−2^.

**Fig. 4:**
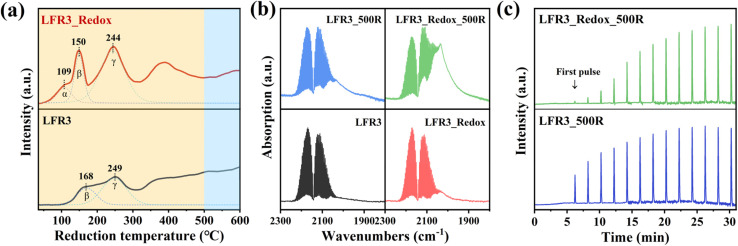
(a) H_2_-TPR profiles of LFR3 and LFR3_Redox, (b) CO-DRIFTS of Ru-containing samples that collected in 2 vol% CO/Ar at room temperature, (c) CO pulse results of LFR3_500R and LFR3_Redox_500R.

The H_2_-TPR is performed from room temperature to 600 °C in order to investigate the reducibility of the Ru incorporated LFR3 and LFR3_Redox samples (*cf.*Fig. 4a). The deconvolution of the H_2_-TPR profiles identifies two reduction peaks for LFR3 at 168 °C (peak β) and 249 °C (peak γ) within the low temperature range (*T* < 300 °C) (*cf.*Fig. 4a, bottom). This temperature range is widely accepted to result from the complete reduction of RuO_2_ to metallic Ru.^41^ However, *in situ* XPS results demonstrate that the complete reduction of oxidized Ru to Ru^0^ does not occur within this temperature range, as evidenced by the absence of Ru^0^ species in LFR3_300R (Fig. 2a). This observation suggests that the peaks observed at 168 °C and 249 °C are due to the formation of oxygen vacancies and the partial reduction of Ru^3+^ to Ru^β^. The broad overlapping H_2_ consumption at higher reduction temperatures is then attributed to the further reduction of Ru^β^ to Ru^0^.

For LFR3_Redox (*cf.*Fig. 4a, top) a novel reduction peak emerges at 109 °C (peak α) in conjunction with the two reduction peaks previously identified in LFR3. This reduction peak cannot be ascribed to the reduction of RuO_2_ to Ru, since there is no Ru^0^ signature discernible in XPS for reduction temperatures below 300 °C (*cf.*Fig. 2b). Consequently, this low-temperature reduction feature is likely associated with the reduction of surface-adsorbed oxygen species.^42^ Additionally, peaks β and γ shift to lower temperatures, 150 °C and 244 °C, respectively, indicating an increased reducibility of LFR3_Redox after the high-temperature redox treatment.^43^ The H_2_ consumption of LFR3_Redox below 300 °C (239 μmol g^−1^) is significantly higher than that of LFR3 (146 μmol g^−1^). This increase in H_2_ consumption is attributed to the enrichment of Ru in the near-surface region induced by the redox pretreatment.

Carbon monoxide (CO) has been shown to adsorb strongly and specifically on Ru, making it an ideal probe molecule for quantifying the exposed surface Ru concentration in the topmost layer.^35^ CO DRIFT spectra are collected during exposure of the Ru-containing perovskites to a flow of 2 vol% CO/Ar at 50 sccm at room temperature. The gas-phase CO concentration remains constant throughout the DRIFTS experiment, enabling the utilization of the CO gas phase Ro-Vi feature (1900–2300 cm^−1^) as a reference for signal normalization. For LFR3 and LFR3_Redox, overlapping absorption bands are observed between 1970 cm^−1^ and 2070 cm^−1^ corresponding to CO adsorbed on Ru at the perovskite surface. The adsorbed CO area is substantially larger for LFR3_Redox than for LFR3, suggesting a higher Ru concentration in the topmost layer, which is consistent with XPS experiments (*cf.*Fig. 2). Upon reduction at 500 °C for 3 hours, Ru in the near-surface exsolves in the form of small particles decorating the surface, which significantly increases the Ru content in the topmost layer. As demonstrated in Fig. 4b, the area of adsorbed CO for both LFR3 and LFR3_Redox is notably augmented upon reduction at 500 °C. The area of adsorbed CO of LFR3_Redox_500R is estimated to be 3.8 times larger than that of LFR3, providing a preliminary estimate of the increase in the number of active sites.

To further quantify the number of active sites, CO uptake experiments are conducted, and the results are summarized in Fig. 4c. The data clearly indicate that LFR3_Redox_500R can adsorb substantially more CO than LFR3_500R. The number of active Ru sites in LFR3_Redox_500R is calculated to be 3.1 times higher than that of LFR3_500R. The resulting Ru dispersion of LFR3_Redox_500 turns out be 26% when the number of active Ru sites is normalized to the total number of Ru in the sample.

**Fig. 5:**
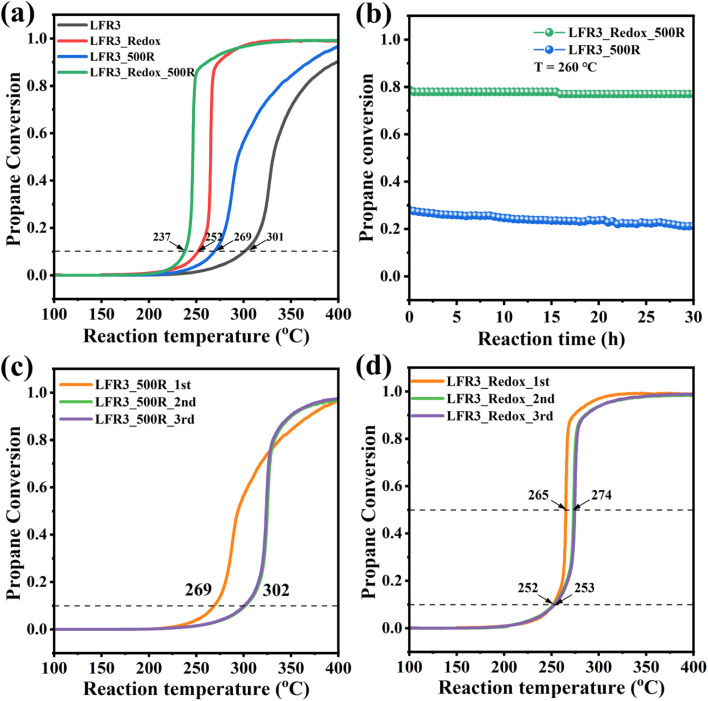
(a) The activity of Ru-containing perovskites in the propane combustion reaction (1 vol% C_3_H_8_, 10 vol% O_2_ and 89 vol% N_2_ at a total flow rate of 100 sscm), (b) the long-term stability of LFR3_500R and LFR3_Redox_500R at 260 °C. Three consecutive activity tests of (c) LFR3_500R and (d) LFR3_Redox_500R.

The propane oxidation activity of the Ru-containing perovskite oxides is evaluated and the corresponding light-off curves and T10 values (temperatures at which 10% propane conversion is achieved, which serves as indicator of catalytic performance) are presented in Fig. 5a. For the LFR3 sample, a maximum propane conversion of 90% is observed at 400 °C, with a T10 value of 301 °C. For the LFR3_500R sample, Ru exsolution further enhances the activity, yielding a T10 value of 269 °C. The catalytic activity of LFR3_Redox is found to be significantly higher than that of LFR3, reaching full conversion within the test condition and a T10 value of 252 °C. Following the exsolution at 500 °C, the LFR3_Redox_500R specimen exhibited a remarkably enhanced catalytic performance (significantly higher than that of LFR3_500R), reducing the T10 value to 237 °C. The STY values of these perovskite samples at 230 °C are measured to be 3.0, 6.3, 14.0, 24.5 mol_CO_2__ h^−1^ kg_cat_^−1^ for LFR3, LFR3_500R, LFR3_Redox and LFR3_Redox_500R, respectively (Fig. S8†). The STY_*n*_ is calculated to compare the intrinsic activity of the two Ru-exsolved samples: LFR3_Redox_500R is determined to have a STY_*n*_ of 0.21 mol_CO_2__ mol_active site_^−1^ s^−1^, which is higher than that of LFR3 _500R (0.17 mol_CO_2__ mol_active site_^−1^ s^−1^). This slight discrepancy can be attributed to the comparatively smaller size of the exsolved particle, as observed in TEM analysis.

The long-term stability of the optimized Ru-containing sample, LFR3_Redox_500R, is assessed at a constant temperature of 260 °C over a duration of 30 hours. As illustrated in Fig. 5b, a negligible decline in the propane conversion from 78% to 77% was observed, indicating satisfactory stability of LFR3_Redox_500R. On the contrary, the propane conversion rate of LFR3_500R under identical reaction conditions gradually declines from 28% to 21% over 30 h on stream. Furthermore, the catalytic activity of LFR3/LFR3_500R and LFR3_Redox/LFR3_Redox_500R decreased only after the first activity test (see Fig. S9† and 5c, d), while no further deactivation is observed in subsequent cycles. Conversely, the light-off curve of LFR3_500R exhibits substantial variation between the first two reaction cycles, with the T10 values of 269 °C and 302 °C, likely attributable to the partial re-incorporation of the exsolved Ru particles. In contrast, the LFR3_Redox_500R exhibits only a slight decrease in the activity between the first two reaction cycles, suggesting that the re-incorporation of the exsolved Ru is largely suppressed.

**Fig. 6:**
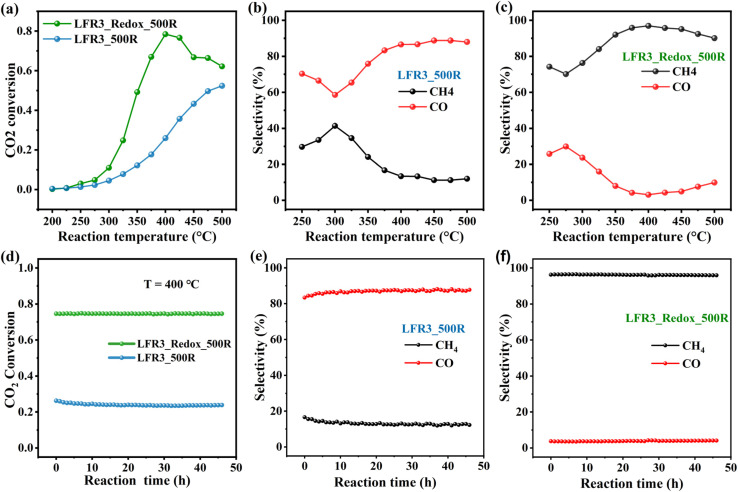
(a) Activity and (b and c) selectivity of CO_2_ hydrogenation over LFR3_500R and LFRO_Redox_500R as a function of temperature. Long-term stability in terms of (d) activity and (e and f) selectivity of LFR3_500R and LFRO_Redox_500R in the CO_2_ hydrogenation reaction at 400 °C. The feeding gas consists of 4 sccm of CO_2_ and 16 sccm of H_2_ that is balanced by 20 sccm of Ar.

The second test reaction is a catalytic reduction reaction, where deactivation due to back dissolution of exsolved Ru particles is not a concern. As reported in the literature, Ru can readily dissociate H_2_, thus rendering it an ideal catalyst for reactions involving H_2_ such as in the CO_2_ hydrogenation reaction.^44^Fig. 6 provides a synopsis of the activity and long-term stability experiments of both Ru-exsolved catalysts in the CO_2_ hydrogenation reaction. The results indicate that LFRO_Redox_500R, with its high surface Ru concentration, exhibits enhanced catalytic performance in comparison to LFR3_500R, attaining a maximum CO_2_ conversion of 78% at 400 °C. Beyond this temperature, a decline is evident in the activity of LFRO_Redox_500R, likely attributable to the attainment of thermodynamic equilibrium. In contrast, no such decline is evident in the activity of LFR3_500R, and the CO_2_ conversion of LFR3_500R increases steadily with rising reaction temperatures, reaching 52% at 500 °C. To elucidate this discrepancy, it is imperative to recall that the formation of methane by CO_2_ hydrogenation is an exothermic reaction (dominant product of LFRO_Redox_500R), while the formation of CO is an endothermic process (dominant product of LFR3_500R).

The product distribution of CO_2_ hydrogenation on the two catalysts is also very different. LFR3_500R leads preferentially to CO formation, *i.e.*, the reverse water gas shift (RWGS) reaction dominates, while for LFRO_Redox_500R methanation is the predominant process. The selectivity of CO_2_ hydrogenation has been shown to depend significantly on the rate of H_2_ activation on supported Ru catalyst as reported in the literature.^45–47^ Consequently, the high Ru-exposed sample LFR3_Redox_500R favors the CH_4_ formation, while the low Ru-exposed sample LFR3_500R prefers the CO formation, aligning with the experimental findings.

During the prolonged tests at 400 °C (see Fig. 6d), LFRO_Redox_500R exhibits remarkable stability over a duration of 45 hours, maintaining a CO_2_ conversion of 76% and a high CH_4_ selectivity of 97%. In contrast, LFR3_500R reveals a modest decline in CO_2_ conversion, decreasing from 26% to 24% within the initial 5 hours, followed by period of stability. Notably, the CO selectivity of LFR3_500R remained consistently high at approximately 90%.

### 3.2 Discussion

The exsolution process of Ru from LFR3 (where 3 at% of Fe is replaced by Ru in LaFeO_3_) has been thoroughly investigated, providing fundamental insights and detailed knowledge.^32,33^ This research has enabled the design of an improved catalyst, characterized by a high concentration of small Ru particles on the surface of the LFR3 perovskite. The high-temperature redox treatment has been shown to increase the concentration of Ru in the near-surface region. The asymmetry of fast exsolution, coupled with the slow redissolution of Ru particles, facilitates the accumulation of ruthenium in the near-surface region of LFR3. The final reduction step at 500 °C enables the production of a high concentration of small particles. The reduction temperature of 500 °C is meticulously selected to facilitate the facile exsolution of ruthenium from the near-surface region without the formation of a passivating LaO_*x*_ layer.

In general, the atomic efficiency of exsolved Ru particles is low because most of the Ru remains in the LFR3 host lattice and is not exsolved into Ru particles participating in the catalytic reaction. For LFR3 in which Ru is homogeneously distributed, only about 18.3% Ru could be exsolved when reduced with H_2_ at 500 °C. However, when the surface region of LFR3 is first enriched with Ru by applying a high-temperature redox pretreatment, approximately 56% of Ru can be exsolved after reduction at 500 °C. This significantly enhances the mass activity of the LFR3 catalyst in the two prototypes of catalytic reactions: the propane combustion (oxidation prototype) and the CO_2_ hydrogenation reaction (reduction prototype). It is noteworthy that LFR3_Redox_500R exhibits superior performance in both catalytic reactions, demonstrating enhanced activity and stability compared to LFR3_500R.

Due to the high-temperature calcination step during the preparation of LFR3, the resulting particles have a mean diameter of approximately 100 nm. Consequently, the diffusion path of internal Ru to reach the surface region is quite extensive, which severely limits the exsolution capacity. The application of redox treatment has been demonstrated to circumvent this limitation; however, it may concomitantly result in structural instability of LFR3.^31^ Therefore, the maintenance of a low concentration of Ru with in LFR3 facilitates the preservation of the bulk structure's stability subsequent to the enrichment of Ru in the near-surface region. This approach is not only instrumental in ensuring the stability of the structure but also in minimizing the quantity of costly Ru utilized.

The following discussion will address the underlying chemical and physical processes responsible for the near-surface accumulation of ruthenium are discussed below. Reduction at temperatures below 500 °C does not enrich the surface Ru concentration at LFR3. However, when the reduction temperature is increased from 500 °C to 800 °C, there is a substantial accumulation of Ru observed near the surface, as evidenced by XPS in our previous work.^33^ The high-temperature reduction step of 800 °C initially causes a deep reduction of the LFR3, which creates diffusion pathways for Ru from deeper layers to the surface. The exsolution process commences at 500 °C, leading to a depletion of Ru concentration in the surface region of the host lattice. This establishes a concentration gradient of Ru from the bulk to the surface, thereby driving the actual diffusion process from the bulk. The second step of the high temperature redox pretreatment, the high oxidation treatment at 800 °C, results in the re-dissolution of the exsolved Ru particles into the perovskite lattice (regeneration). This step is in competition with the reoxidation of LFR3. Due to the short diffusion paths, Ru can rapidly dissolve back into the near-surface region. However, the reoxidation of the LFR3 sample hinders the deeper penetration of Ru back into the bulk of LFR3, resulting in most of the Ru remaining in the near surface region. This enrichment is evidently observed by XPS (see Fig. 2).

The final mild reduction step, carried out at a modest temperature of 500 °C, is employed to facilitate the exsolution of the Ru that has accumulated in the near-surface region of LFR3_Redox, thereby forming small Ru particles at the surface. At a reduction of 500 °C, the exsolution of near-surface ruthenium is quite efficient, as demonstrated in our previous study, while further bulk diffusion of Ru is suppressed.^33^ The selected temperature of 500 °C is also low enough to suppress the passivation of the Ru particles by a covering LaO_*x*_ layer.^32^ The passivation layer begins to form at 600 °C.^33^

The density and size of the exsolved particles can be explained by nucleation and growth.^45,48,49^ The Ru enrichment in the near-surface region of LFR3_Redox leads to a high Ru flux towards the surface during the exsolution process. Since the reduction temperature of 500 °C is modest, the critical nucleus of Ru is small. Consequently, a greater number of smaller Ru particles are formed at the LFR3_Redox surface, presumably at structural defects on the surface of the host material; a similar behavior was recently reported for a related system, the Ir exsolution from Ir doped SrTiO_3_.^27^ For LFR3, the lower concentration of Ru in the near-surface region results in a much lower flux of Ru to the surface and consequently in a lower density of critical nuclei. The reduced density of critical nuclei and the reduced influx of Ru on LFR3 relative to LFR3_Redox leads to the preferential growth of particles rather than the formation of more stable (critical) nuclei. This phenomenon naturally explains the lower density of Ru particles on LFR3_500R, but also the slightly larger size of the resulting exsolved particles on LFR3_500R (2.3 ± 0.4 nm) if compared to LFR3_Redox_500R (1.9 ± 0.3 nm). The particle density of LFR3_500R is estimated to be 4350 μm^−2^ (as determined by STEM, see Fig. 3), while the density of LFR3_Redox_500R is 26 000 μm^−2^. For comparison, the particle density of LFR3_800R is estimated to be 133 μm^−2^ with a mean particle size of 12 nm (Fig. S3†).

In this study, we examined the catalytic performance of two catalysts, LFR3_500R and LFR3_Redox_500R, in two distinct catalytic reactions. The first reaction involved the total oxidation of propane, while the second reaction involved the reduction of CO_2_ by hydrogenation. Our findings revealed that LFR3_Redox_500R exhibited significantly enhanced catalytic performance compared to LRF3_500R in both reactions. The conclusion drawn from the long-term stability in both catalytic reactions is that the designed catalyst LFR3_Redox_500R exhibits enhanced stability, surpassing that of LFR3_500R.

## 4 Conclusions

In order to enhance the atomic efficiency of exsolved particles from a 3 at% Ru-substituted LaFeO_3_ perovskite oxide (LFR3), a rational catalyst synthesis protocol has been developed. This design involves tuning the exsolution process in a reducing atmosphere at 500 °C and enriching the Ru concentration in the near-surface region of LFR3 by subjecting a high-temperature redox pretreatment at 800 °C. This pretreatment results in a catalyst LFR3_Redox_500R with a much higher concentration of small Ru particles (26 000 μm^−2^, 1.9 ± 0.3 nm) and thus a much higher mass activity than the untreated catalyst LFR3_500R (4350 μm^−2^, 2.3 ± 0.4 nm). The LFR3_redox_500R catalyst exhibits superior performance in two prototypical reactions, the catalytic propane combustion and the CO_2_ hydrogenation reaction under strongly oxidizing and reducing reaction conditions, respectively. In both types of reactions, the LFR3_redox_500R demonstrates enhanced stability and activity compared to LFR3_500R. In summary, a superior Ru-based catalyst has been prepared and designed based on the fundamental understanding of the exsolution process of LFR3.

## Data availability

The original data supporting this article are available in the main context and ESI.†

## Author contributions

YW, JG, and HO conceptualized and designed the project. Data acquisition and analysis were conducted by YW, PP, LP, AS, LG, AX, ZH, MD, and WH. The project was supervised by YG, BS, and HO. YW drafted the original manuscript and HO reviewed and edited it.

## Conflicts of interest

There are no conflicts to declare.

## Supplementary Material

SC-OLF-D5SC00778J-s001
