# Supplementary material for: Boosting Ru atomic efficiency of LaFe0.97Ru0.03O3via knowledge-driven synthesis design
Source: Chem Sci. 2025 Mar 28;16(18):7739–50. doi: 10.1039/d5sc00778j (PMC11973924; doi:10.1039/d5sc00778j)
Supplement: SC-016-D5SC00778J-s001 [file SC-016-D5SC00778J-s001.pdf]

Supplementary Information (SI) for Chemical Science.
This journal is © The Royal Society of Chemistry 2025

Supporting Information

Boosting Ru atomic efficiency of $LaFe_{0.97}Ru_{0.03}O_3$ via a knowledge-driven synthesis design

Yu Wang[a,b], Paul Paciok[c], Lukas Pielsticker[d], Alexander Spriewald Luciano[b], Lorena Glatthaar[b], Zimo He[a], Min Ding[a], Walid Hetaba[d], Jaime Gallego[b,e], Yanglong Guo[a]*, Bernd M. Smarsly[b,e]*, Herbert Over[b,e]*

[a] *State Key Laboratory of Green Chemical Engineering and Industrial Catalysis, Research Institute of Industrial Catalysis, School of Chemistry and Molecular Engineering, East China University of Science and Technology, Shanghai 200237, PR China*
[b] *Institute of Physical Chemistry, Justus Liebig University, Heinrich-Buff-Ring 17, D-35392 Giessen, Germany*
[c] *Ernst Ruska-Centre for Microscopy and Spectroscopy with Electrons and Peter Grünberg Institute, Forschungszentrum Jülich GmbH, Jülich, 52425, Germany*
[d] *Department of Heterogeneous Reactions, Max Planck Institute for Chemical Energy Conversion, Stiftstr. 34-36, 45470 Mülheim an der Ruhr, Germany*
[e] *Center for Materials Research, Justus Liebig University, Heinrich-Buff-Ring 17, D-35392 Giessen, Germany*

* Corresponding authors:
Herbert.Over@phys.Chemie.uni-giessen.de;
Bernd.Smarsly@phys.Chemie.uni-giessen.de;
ylguo@ecust.edu.cn;

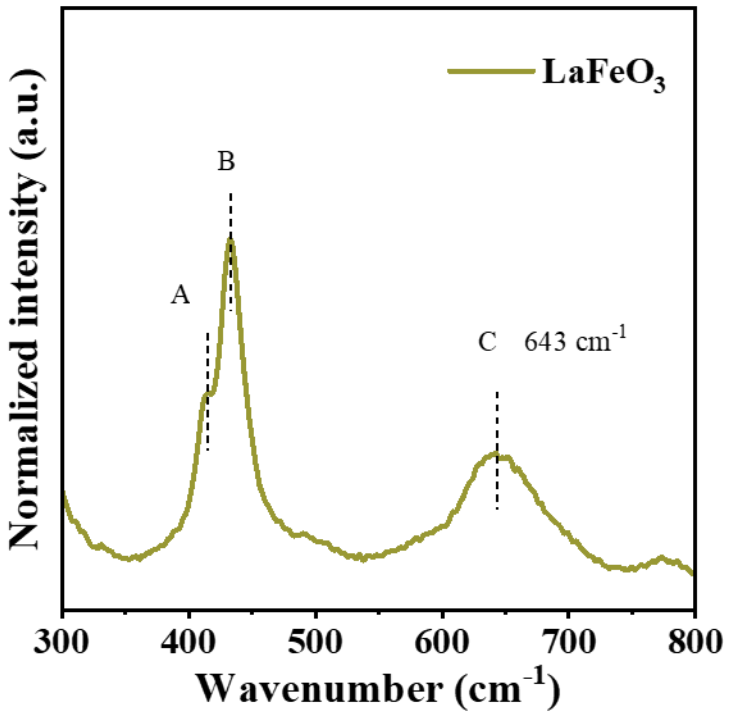

Figure S1. Raman spectra of $LaFeO_3$.

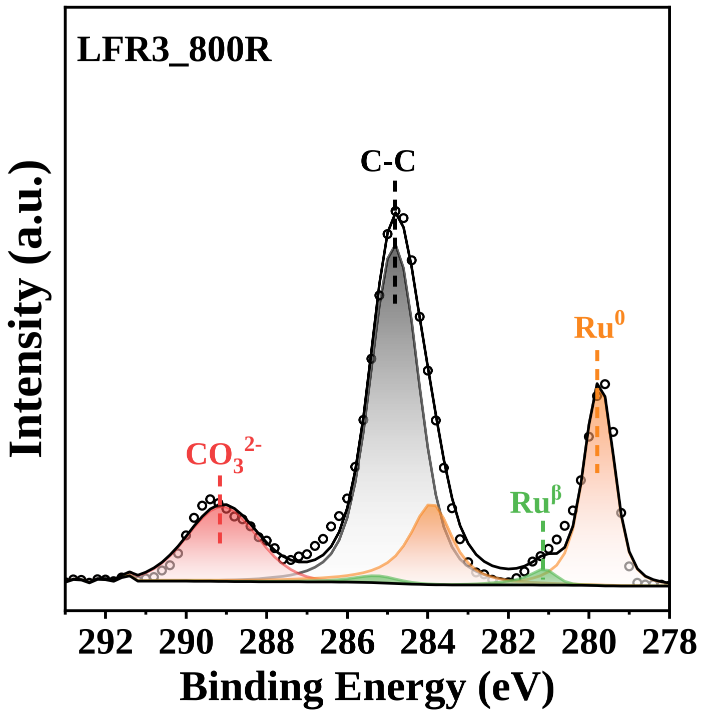

Figure S2. Fitted C 1s + Ru 3d XP spectrum of LFR3_800R.

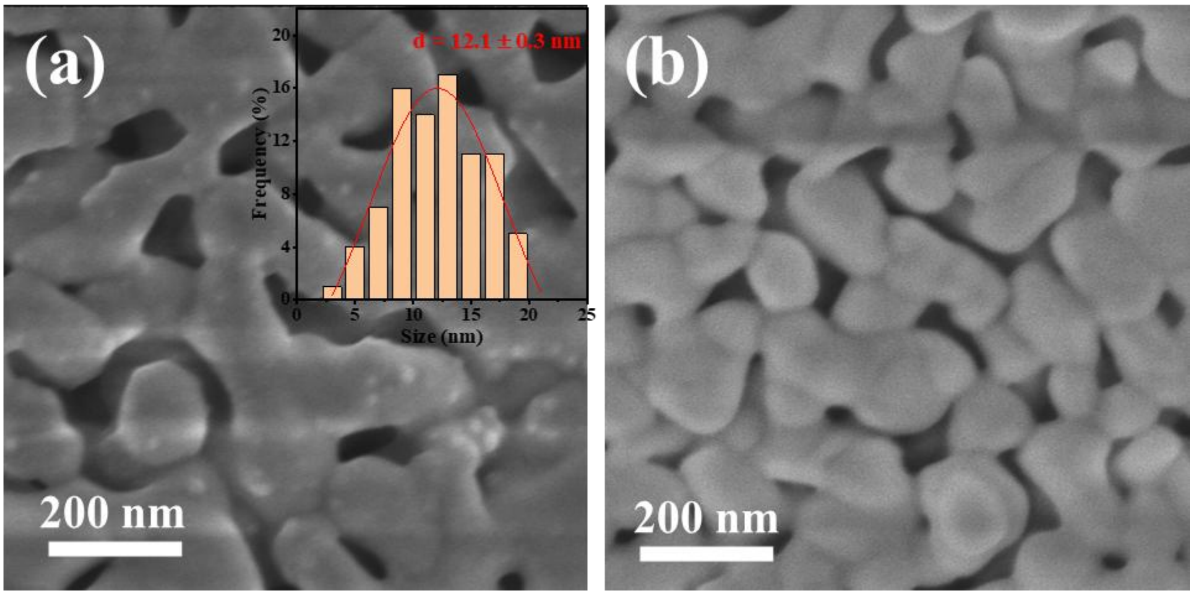

Figure S3. SEM pictures of (a) LFR3_800R and (b)LFR3_Redox (LFR3_800R_800O). The particle size distribution of LFR3_800R is provided in the inserted picture.

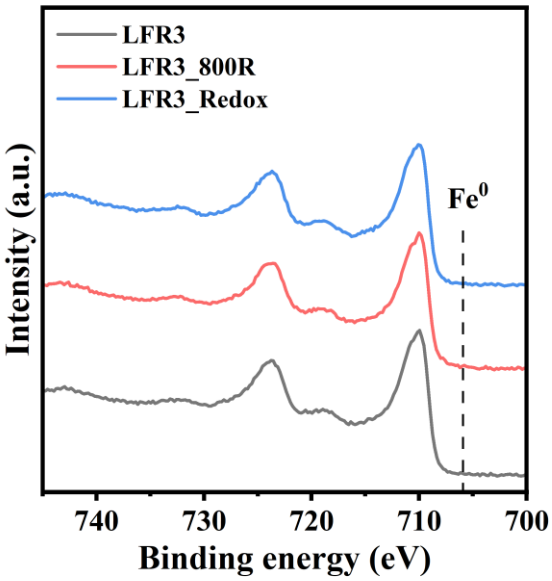

Figure S4. Fe 2p XPS spectra of LFR3, LFR3_800R and LFR3_Redox.

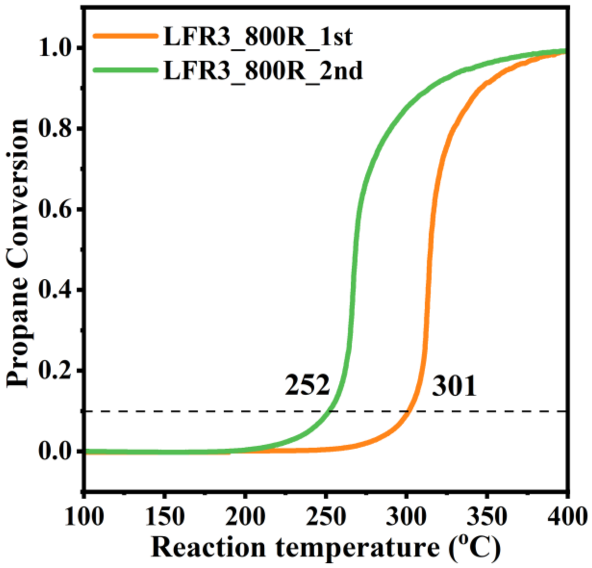

Figure S5. Consecutive propane oxidation reaction cycles of LFR3_800R.

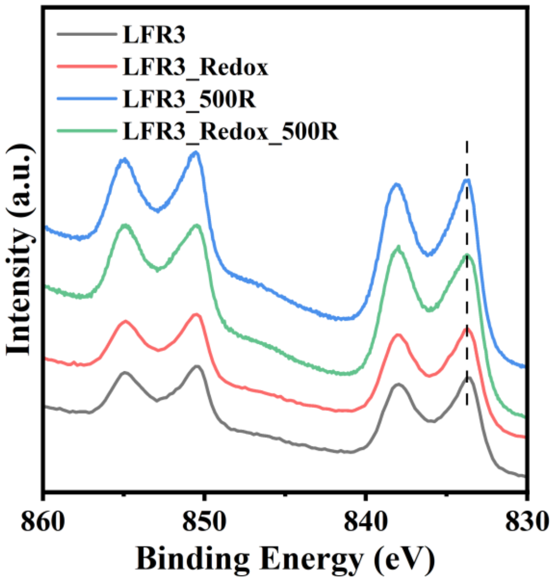

Figure S6. La3d XPS spectra of Ru-doped samples LFR3, LFR3_Redox and Ru exsolved Ru samples LFR3_500R, LFR3_Redox_500R.

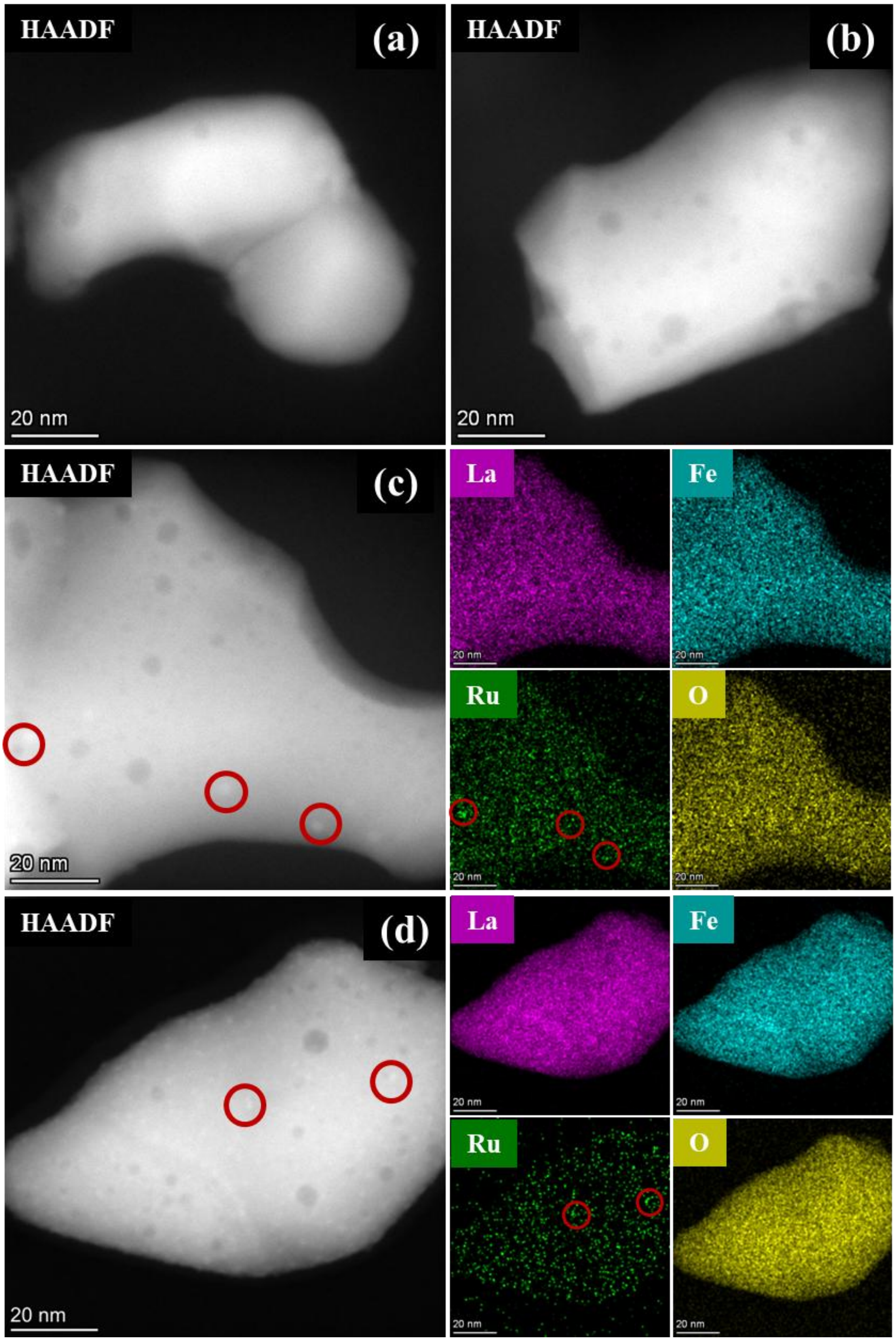

Figure S7. HAADF images of (a) LFR3, (b) LFR3_Redox, (c) LFR3_500R and (d) LFR3_Redox_500R. The corresponding EDS-Mappings of the Ru-exsolved samples are presented with the red circles to show some exsolved Ru particles.

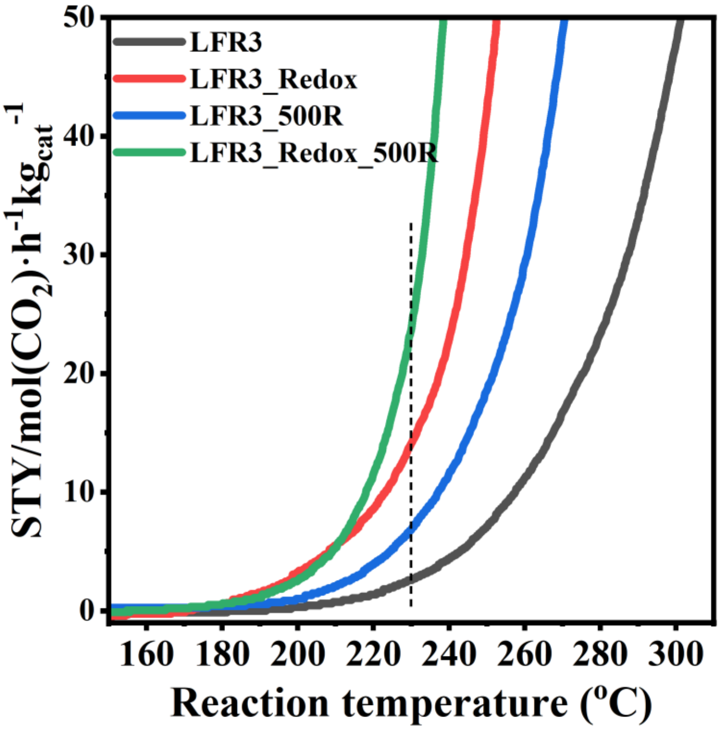

Figure S8. The STY of LFR3, LFR3_Redox, LFR3_500R, LFR3_Redox_500R as a function of reaction temperature. The STY values at 230 °C are used to compare the activity.

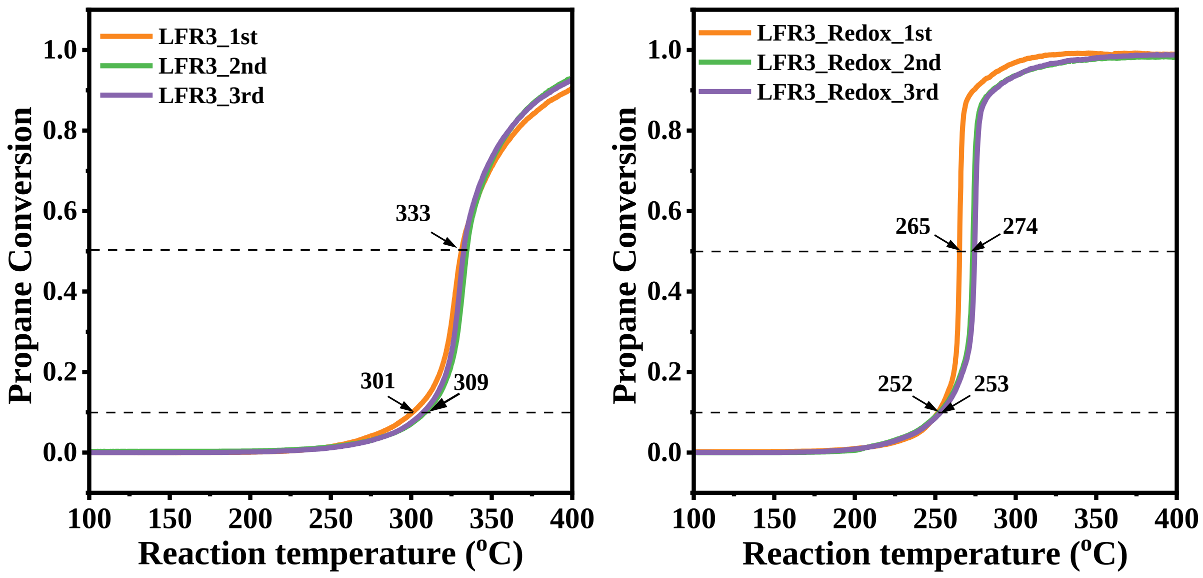

Figure S9. Three consecutive propane oxidation reaction cycles of LFR3 and LFR3_Redox.

Table S1. The Ru 3d fitting parameters of LFR3 in the *in-situ* XPS experiments.

	Ru^{3+} $3d_{5/2}$		Ru^{3+} $3d_{3/2}$		Ru^{β} $3d_{5/2}$		Ru^{β} $3d_{3/2}$		Ru^{0} $3d_{5/2}$		Ru^{0} $3d_{3/2}$	
Line shape	LF(0.4,1,45,280)		LF(0.8,1,45,280)		LF(0.4,1,45,280)		LF(0.6,1,45,280)		LF(0.8,1,45,280)		LF(1.01,1.25,500,50)	
	BE (eV)	FWHM	BE (eV)	FWHM	BE (eV)	FWHM	BE (eV)	FWHM	BE (eV)	FWHM	BE (eV)	FWHM
LFR3	282.3	1.60	286.5	2.20	281.2	1.40	285.4	2.20	-	-	-	-
_600O	282.3	1.22	286.4	1.46	281.1	0.95	285.3	1.14	-	-	-	-
_300R	282.3	1.40	286.4	1.68	281.1	1.32	285.3	1.60	-	-	-	-
_400R	-	-	-	-	281.0	1.25	285.2	1.62	-	-	-	-
_500R	-	-	-	-	281.1	1.19	285.3	1.43	280.1	1.17	284.3	1.31

Table S2. The Ru 3d fitting parameters of LFR3_Redox in the *in-situ* XPS experiments.

	Ru^{3+} $3d_{5/2}$		Ru^{3+} $3d_{3/2}$		Ru^{β} $3d_{5/2}$		Ru^{β} $3d_{3/2}$		Ru^{0} $3d_{5/2}$		Ru^{0} $3d_{3/2}$	
Line shape	LF(0.4,1,45,280)		LF(0.8,1,45,280)		LF(0.4,1,45,280)		LF(0.6,1,45,280)		LF(0.8,1,45,280)		LF(1.01,1.25,500,50)	
	BE (eV)	FWHM	BE (eV)	FWHM	BE (eV)	FWHM	BE (eV)	FWHM	BE (eV)	FWHM	BE (eV)	FWHM
LFR3_Redox	282.3	1.46	286.5	1.80	281.2	1.31	285.4	2.00	-	-	-	-
_600O	282.3	1.69	286.5	2.03	281.1	1.50	285.3	1.80	-	-	-	-
_300R	282.3	1.24	286.4	1.49	281.1	1.86	285.3	2.64	-	-	-	-
_400R	-	-	-	-	281.1	1.59	285.3	1.94	280.1	1.2	284.3	1.68
_500R	-	-	-	-	281.1	1.47	285.3	1.99	280.1	1.51	284.3	1.80
